# Cell Death and Transcriptional Responses Induced in Larvae of the Nematode *Haemonchus contortus* by Toxins/Toxicants with Broad Phylogenetic Efficacy

**DOI:** 10.3390/ph14070598

**Published:** 2021-06-22

**Authors:** Douglas P. Jasmer, Bruce A. Rosa, Makedonka Mitreva

**Affiliations:** 1 Department of Veterinary Microbiology and Pathology, Washington State University, Pullman, WA 99164, USA; djasmer@wsu.edu; 2 Department of Medicine, Division of Infectious Diseases, Washington University School of Medicine, St. Louis, MO 63110, USA; barosa@wustl.edu; 3 Department of Genetics, Washington University School of Medicine, St. Louis, MO 63110, USA; 4 McDonnell Genome Institute, Washington University School of Medicine, St. Louis, MO 63108, USA

**Keywords:** nematode, intestine, anthelmintics, microscopy, pathology, RNA-seq

## Abstract

Establishing methods to investigate treatments that induce cell death in parasitic nematodes will promote experimental approaches to elucidate mechanisms and to identify prospective anthelmintics capable of inducing this outcome. Here, we extended recent progress on a method to monitor cell death and to identify small molecule inhibitors in *Ascaris suum* to *Haemonchus contortus*, a phylogenetically distant parasitic nematode of significance for both human and agricultural animal health. We utilized a diverse group of small molecule inhibitors referred to as nematode intestinal toxins/toxicants (NITs) coupled with motility, cytological and cell death assays to resolve gross effects on motility and individual cells and organ systems of two *H. contortus* larval stages in culture. Early transcriptional response evaluation identified NIT-responsive genes and pathways. The scope of death among cells in larvae varied among NITs but shared patterns with *A. suum*, despite the approach having some limitations due to characteristics of *H. contortus* larvae. Gene response patterns varied among NITs tested and provided information on the cell targets and pathways affected. Experimental NIT assays provide tools capable of inducing cell death in larval stages of parasitic nematodes, and can resolve many individual cells and organ systems in which cell death can be induced.

## 1. Introduction

Parasitic nematodes cause diseases that produce substantial mortality and morbidity among humans across the globe. These pathogens also reduce food production in livestock and plants that, in turn, differentially restricts the nutritional resources of the global population with the lowest incomes. Anthelmintic treatments are heavily relied upon in efforts to control and treat infections caused by nematode pathogens. Impeding these efforts is the limited selection of efficacious anthelmintics coupled with emerging anthelmintic resistance among these pathogens [1]. While the situation identifies a need to expand the number and kind of available anthelmintics, technical advances that enhance research capabilities are also needed to accelerate the accomplishment of this goal. 

To advance research on anthelmintics and experimental capabilities on nematode pathogens, we established an approach with a design for pan-Nematoda applications that has a primary focus on a single-parasite tissue, the intestine. The rationale for this focus relates mainly to its apparent hypersensitivity to some anthelmintics and relative tractability to experimental approaches compared with other tissues, as described elsewhere [2]. Capitalizing on a pan-Nematoda multiomics database for the intestine, combined with other biologic and pharmacological considerations, we experimentally derived a set of drug-like molecules referred to as nematode intestinal toxins/toxicants (NITs). Each NIT caused pathology in intestinal cells of larval stages and, in some cases, adult worms of *Ascaris suum*, a model species to study the intestine of parasitic nematode species [3]. The identification of potential inhibitors of secretory processes was the organizing principle for the *de novo* identification of NITs, and multiple NITs demonstrated efficacy against phylogenetically diverse nematode species representing whipworms (Clade I), filarial worms (Clade III), and *C. elegans* (Clade V) [3]. 

Cellular proteins annotated as targets for each NIT collectively identify a diverse set of potential target proteins of significance in *A. suum* intestinal cells [4]. Although new approaches to kill nematode intestinal cells was a goal of this research, expression in other tissues was expected for many of the potential target proteins. In this context, an approach was developed to rapidly determine the range of cells induced to undergo cell death by experimental treatments, such as NITs, in whole *A. suum* larvae in culture. Information of this kind can augment that obtained for prospective anthelmintics identified by using motility assays. The assay utilized propidium iodide (PI), a DNA-binding vital dye to detect the end point of cell death in whole nematodes in culture. Assignment of death to specific cells and organs was facilitated by anatomic details established by using a combination of the cell permeable DNA-binding dye, bisbenzimide (BB), and differential interference contrast (DIC) microscopy. The method rapidly resolved cell death in numerous cells/organs (e.g., intestine, nervous system, excretory system, seam cells, hypodermal cells, and others) of live larvae while in culture, and the results highlighted differences among cell death profiles induced by NITs. Thus, NITs represent tools to investigate cellular mechanisms leading to death in many cells of parasitic nematodes, and the PI cell death assay provides a read out for related experimental manipulations in this context. Related applications have been achieved with the non-parasitic nematode *Caenorhabditis elegans* [5,6]. 

Components of early gene response patterns (GRPs) induced by NITs may reflect elements of pathways involved with initiating or mediating cell death responses. RNA-seq analysis following treatment with NITs identified early GRPs in *A. suum* larvae that informed on potential target proteins and pathways (e.g.,dihydroorotate dehydrogenase (uridine synthesis) and RAB GTPase(s) (vesicle transport)) that may relate to initiator events leading to cell death induced by selected NITs [4]. Information of this kind can aid in the design of experiments to manipulate cell death outcomes initiated by NITs and assessed by the PI cell death assay. 

The overall design of our larger research effort was intended to advance knowledge in a pan-Nematoda context involving three phylogenetically diverse parasitic species, each with distinct evolutionary pathways to parasitism (*Haemonchus contortus*, clade V; *A. suum*, clade III; and *Trichuris suis*, clade I). These three species were used for the initial computational prioritization of NITs [3], and they collectively represent much of the phylogenetic distance in the Nematoda, which includes parasites of many species including humans, animals, and plants (where parasitism is defined as requiring a host to complete at least one stage of its life cycle, as opposed to non-parasitic “free-living” species such as *C. elegans* which does not infect any hosts [7]). Although *A. suum* figured centrally in the development of this pan-Nematoda approach [3,4], we here sought to determine if NITs and the PI cell death assay have applications to rapidly detect death induced by NITs in cells and organs of H. contortus, leaving *T. suis* the only one of the three for which applications have yet to be investigated. *H. contortus*, an abomasal parasite of small ruminants, was chosen due to its veterinary importance and a number of experimental advantages it offers, including availability of large numbers of infective L3 and the ability to culture exsheathed L3 (xL3) to “parasitic” L4s. *H. contortus* feeds on blood and is one of the most detrimental pathogens of small ruminants, which are important resources for nutrition and commerce in many developing regions of the world [8,9,10]. The propensity of *H. contortus* to rapidly acquire anthelmintic resistance makes advances to control this parasite highly important. Thus, new tools and biological knowledge related to cellular effects of experimental toxins may promote advances in anthelmintic research on *H. contortus*. Although PI has been used in a general application to monitor anthelmintic effects on *H. contortus* larvae [11], resolution of individual cells and organs relative to cell death outcomes and nuances of a system to do so have not. 

In research reported here, we sought to determine if NITs are toxic to *H. contortus* larval stages, if they cause cell death detectable by the PI assay, the identity of cells and organs that are susceptible to NIT-induced cell death, and the GRPs affected early by selected NIT treatments. The results indicated that, even with limitations stemming from interspecies biological differences, the approach has applications to *H. contortus*, providing new pathological and molecular tools that can be integrated into research on this model species, the progress of which will contribute to the study of nematode pathogens of humans. 

## 2. Results

Results from a PI-based cell death assay and GRPs can augment insights provided by whole-worm motility inhibition assays that are commonly used to evaluate effects of drugs and drug-like compounds on parasitic nematodes. The rapid resolution of individual cells and organs affected by these treatments in live worms greatly enhances details of likely relevance to the anthelmintic effects at play, as was demonstrated in our previous studies in the nematode clade III parasite, *A. suum* [3,4]. In this investigation, we explored the utility of the overall experimental approach to another parasitic species, *H. contortus*, a clade V parasite. That system involves the use of (i) fluorescent nuclear probes (propidium iodide and bisbenzimide), (ii) an *H. contortus* larval culture system, and (iii) RNA-seq GRPs to treatments. The system was demonstrated using our set of six (seven, with this research) small-molecule inhibitors recently demonstrated to target the nematode intestine (nematode intestinal toxins/toxicants, NITs [3]).

### 2.1. Fluorescent Probes Demonstrate Larval Ingestion

**Figure 1:**
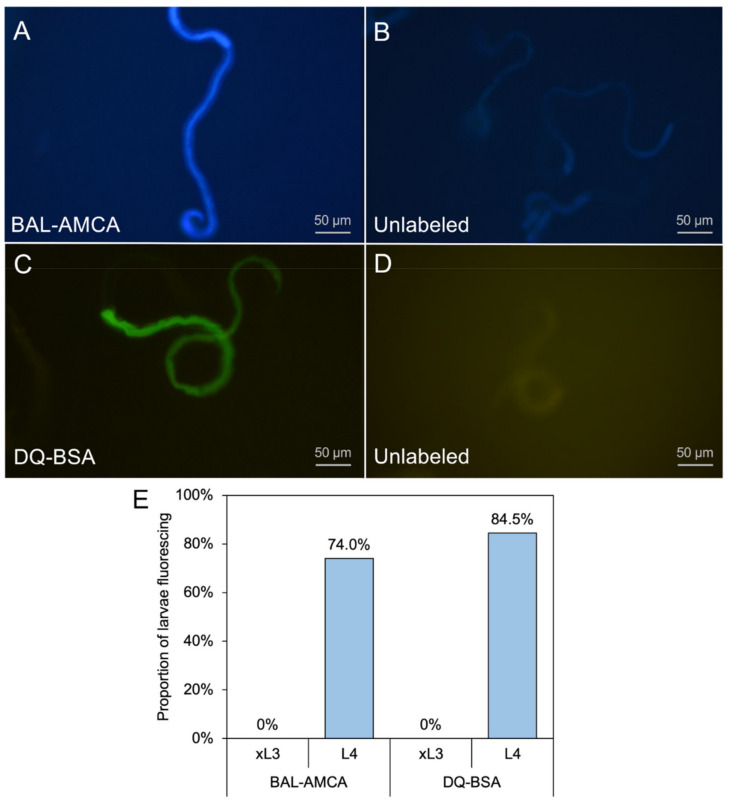
Fluorescent probes demonstrate larval ingestion in L4 *H. contortus*. Beta-ala,lys-AMCA (BAL-AMCA) (**A**), or DQ-Bovine Serum Albumin (DQ-BSA) (**C**) labeled larvae shows probe staining in the intestine. Unlabeled controls (**B**,**D**), respectively) larvae lack staining. (**E**) The proportion of xL3 and L4 larvae with staining by each probe after 4 h incubation.

Two different function-based fluorescent probes were utilized to assess ingestion by *H. contortus* xL3 and L4 larvae: (1) beta-ala,lys-AMCA (BAL-AMCA), a fluorescent dipeptide that enters nematode intestinal cells by receptor-mediated transport [12] (Figure 1A,B); and (2) DQ-Bovine serum albumin (DQ-BSA), a protease substrate that is fluorescence quenched in the intact construct and becomes visible upon proteolytic hydrolysis [13] (Figure 1C,D). We found that a mean 74.0% and 84.5% of *H. contortus* L4, respectively, were positive for signal in the intestinal tract after 4 h when cultured with these probes, while neither probe was detected in the intestines of xL3s (Figure 1E), confirming differences in competence to feed by each of these respective stages under conditions utilized in these experiments. 

### 2.2. NIT Effects on H. contortus Motility

**Figure 2:**
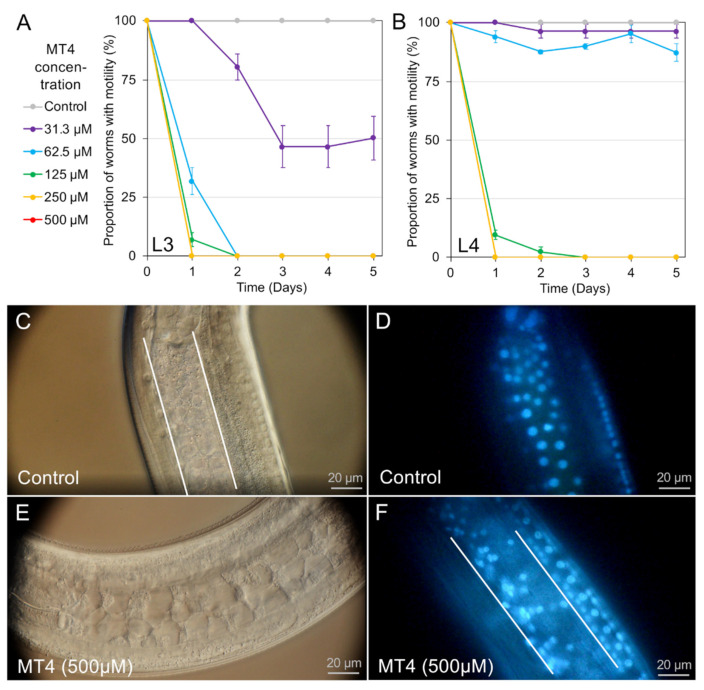
MT4 treatment on *A. suum* larvae. Motility assay results are shown for (**A**) L3-stage larvae (IC_50_ 55.5 μM at 24 h) and (**B**) L4-stage larvae (IC_50_ 91.4 μM at 24 h). (**C**) 2-day untreated control L3 larvae viewed with 60× differential interference contrast (DIC) microscopy (left) shows intact intestinal cells and (**D**) BB nuclear-staining shows a normal pattern of intestinal nuclei. Treatment with MT4 (500 μM) for 2 days induces (**E**) visible destruction of intestinal cells and (**F**) disrupted patterning of intestinal nuclei.

In past research, the effects of six nematode intestinal toxins/toxicants (NITs) were reported for *A. suum* L3 and L4 larvae (staurosporine, ruxolitinib, leflunomide, sunitinib, alvocidib, and CID1067700) [3,4]. A seventh NIT not previously reported (p38 MAP kinase inhibitor IV, also known as MT4) was identified through the same de novo process as in Jasmer et al., 2020 [3] and is a prospective inhibitor of p38 MAP kinases. MT4 showed activity against *A. suum* L3 and L4 in motility assays (Figure 2) and had an IC_50_ of 55.6 µM after 24 h in L3, and 92.2 µM after 24 h in L4. Similar to other NITs, MT4 caused damage to *A. suum* L3 intestinal cells observable by DIC microscopy in which disruption of intestinal cells and cell membranes was evident. Live larvae were labeled with a cell-permeable DNA-binding dye (bisbenzimide, BB) to visualize intestinal cell nuclei, and BB-labeled intestinal cell nuclei in whole L3 showed a disrupted pattern consistent with cell membrane damage. Thus, MT4 meets our definition of a NIT (>70% inhibition at day 4 with the highest dose [3]), coupled with the demonstrated pathology observed in intestinal cells. Because of its relative effectiveness compared to other NITs, MT4 it was included as a seventh NIT in experiments conducted with *H. contortus*.

**Figure 3:**
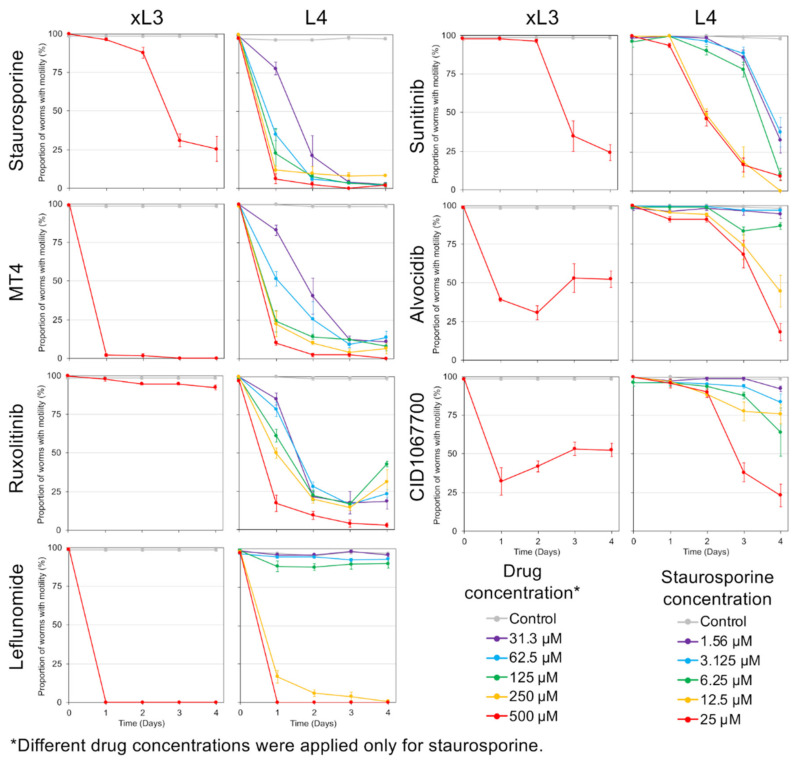
Motility assay results for *H. contortus* xL3 (single concentration) and L4 (dilution series) nematode intestinal toxins/toxicants (NIT) treatments, including staurosporine (IC_50_ of 2.37 µM, 1 day), MT4 (56.7 µM, 1 day), ruxolitinib (221.5 µM, 1 day), leflunomide (L4-stage IC_50_ 192.9 µM after 1 day), sunitinib (161.5 µM, 2 days), alvocidib (427.0 µM, 3 days), and CID1067700 (215.1 µM, 4 days).

Each of the seven NITs was initially tested with a single concentration on xL3 and L4. In general, xL3s showed less sensitivity than L4s to all of the NITs at the highest concentrations used for each, with the exceptions of MT4 which reached full efficacy faster in the xL3 stage, and leflunomide which was 100% effective after 1 day in both stages (Figure 3). Because xL3s are developmentally positioned at the transition to the parasitic phase of the life cycle, our efforts focused to determine IC_50_ values for L4, which, although produced in vitro, are expected to better represent the parasitic phase of the life cycle (Figure 3). Each NIT caused a significant decrease of motility for *H. contortus* L4 at some concentration within the range tested, and the relative effectiveness was ordered according to IC_50_ (Figure S1). As with *A. suum*, staurosporine was by far the most effective, with an IC_50_ of just 2.37 µM after 1 day, and the testing being performed at a lower concentration compared with the other NITs. The newly-added NIT MT4 was the second-most effective, with an IC_50_ of 56.7 µM after 1 day, followed by ruxolitinib with an IC_50_ of 221.4 µM after 1 day, but dropping below 31.3 µM by day 2. As with *A. suum* [3], leflunomide was highly effective at higher concentrations (>250 µM) but was not very effective at lower concentrations. Sunitinib only became effective after 4 days of treatment, while alvocidib and the intestinocentric CID1067700 did not show substantial effects on motility in this assay. In general, these results extended the application of these NITs to include *H. contortus* xL3 and/or L4 relative to research on cellular mechanisms and targets responsible for anthelmintic effects on them.

### 2.3. Live H. contortus xL3 and L4 Nuclei Localization by Bisbenzimide (BB) Labeling

**Figure 4:**
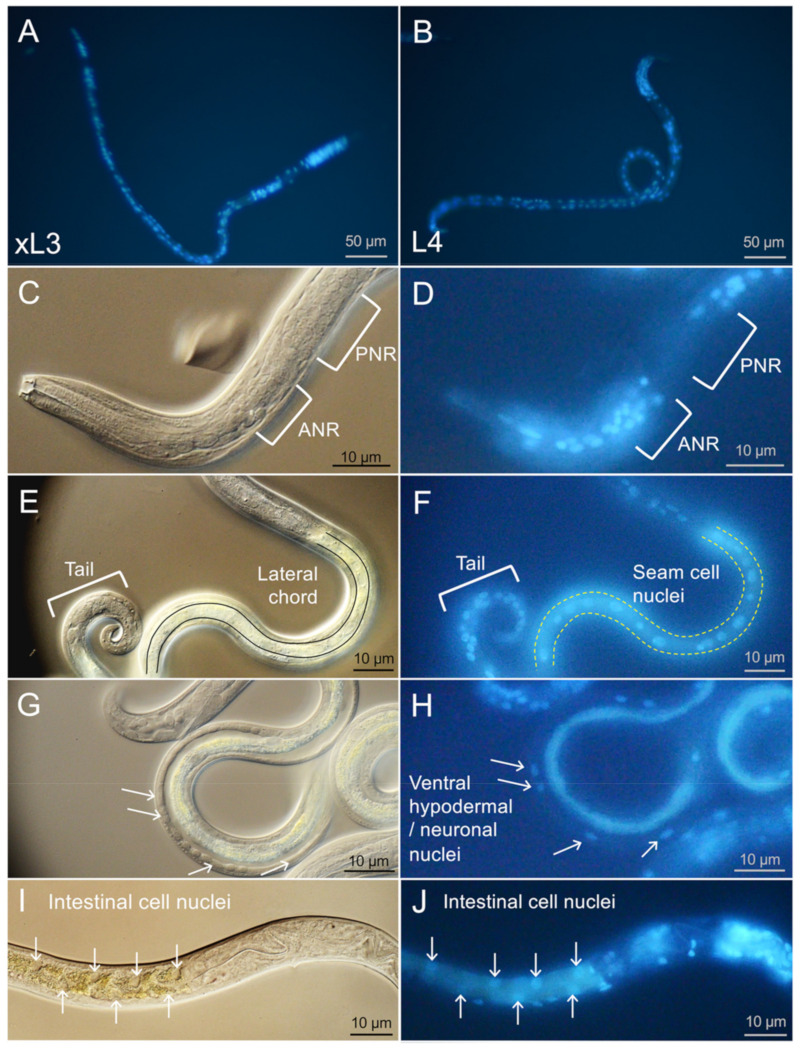
Bisbenzimide (BB) nuclear staining resolves tissues and cells in *H. contortus* larvae. xL3 (**A**) and L4 (**B**) larvae have similar BB staining profiles across the worm. L4 larval nuclei anterior to the nerve ring (ANR) visible by DIC (**C**) stain with BB (**D**), while most of those posterior to the nerve ring (PNR) do not stain, despite the presence of cells. Along the length of the lateral chord to the tail visible by DIC (**E**), seam cell and tail cell nuclei are visible by BB (**F**). Cells visible on the lateral side of the larva by DIC (**G**) contain several ventral hypodermal/neuronal nuclei stained with BB (**H**). Staggered intestinal cell nuclei visible by DIC (**I**) are visible by BB (**J**). Panel A is viewed with a 20× objective (200× magnification), and the remaining panels (L4 larvae) are viewed with 60× objective (600× magnification) and cropped/magnified digitally to highlight features. BB was incubated for 4 h pre-treatment and was included throughout the incubation period. DIC images have been adjusted for brightness and contrast individually to best highlight features of interest.

As with *A. suum*, we labeled live larvae with BB to monitor nuclei localization in healthy and NIT-treated larvae. The ability of BB to penetrate and label nuclei of live xL3 and L4 was first determined for larvae in culture wells (96 well plate) and viewed using a 20× objective (200× magnification) on an inverted microscope. The images showed larvae (Figure 4A and Figure 4B) with a subset of nuclei that were clearly labeled with BB, whereas it was also evident that many nuclei went unlabeled (clarified in the next section). Because of this incomplete staining profile, the images were taken after 16 h exposure to BB in culture in attempts to maximize staining. Periodic observation over longer periods of time did not show any improvement on this pattern of staining. The general pattern of BB nuclear staining was similar for live xL3 and L4. Because of the relatively small size of *H. contortus* larvae (approximately 1/2 to 1/3 of the length and width of *A. suum*) and the incomplete BB staining profile, it was difficult to assign stained nuclei more specifically to organs or cells based on information gained at this magnification. 

Further specificity on the assignment of BB labeled nuclei was gained using DIC coupled with fluorescent microscopy at 600× magnification (Figure 4C–J). In general, DIC resolution of tissues and cells was compromised for *H. contortus* larvae over much of the body posterior to the esophagus due in part to refractive granules located predominantly in intestinal cells. For reasons that are unclear, intact *H. contortus* larvae also often produced relatively poor contrast under DIC conditions used here, impeding the resolution of cells. Despite these two limitations, a range of cells could be discerned, including cells and nuclei located where neurons have been documented anterior and posterior to the nerve ring in *H. contortus* and/or *C. elegans* research [14,15], lateral chords with associated seam cells (multipotent lateral hypodermal cells which lie along each side of the worm [16]; Figure 4E,F), ventrally located nuclei that represent presumptive hypodermal and neuronal nuclei associated with the ventral chord (Figure 4G,H), the intestine (Figure 4I,J), and cells comprising the posterior tail region (Figure 4E, likely to include hypodermal, neuronal, muscle nuclei), as is found in *C. elegans*. The ventral location of apparent hypodermal nuclei is similar to *C. elegans* [17], but different from A. suum larvae in which they associate with the lateral chord [4].

Following structural clarification from DIC examination, prominent areas and cells that were routinely observed with BB labeling (results only shown for L4) included nuclei anterior to the nerve ring, cells in the lateral chord (presumptive seam cells), and cells in the posterior tail region of larvae. Nuclei from prominent organs in which BB labeling was routinely absent, or more variable, included those of apparent neurons posterior to the nerve ring, intestinal cells, esophageal cell, muscle cells, and most ventral chord nuclei (Figure 4). The BB-labeled intestinal cell nuclei shown in Figure 4J were an uncommon finding and were included to show the staggered appearance of those nuclei along the longitudinal axis of the larvae (useful for assessing cell death, see next section). BB staining of ventral chord nuclei was more sporadic with many often unstained by BB (Figure 4H). Features necessary to differentiate between the ventral neuronal and hypodermal nuclei were not evident with available details, and thus the variability could reflect preferential staining of either cell type or combinations of both. 

To summarize, DIC microscopy coupled with BB labeling aided in resolution of many nuclei, cells, and organ systems in *H. contortus* xL3 and L4. The observations provide a baseline to better interpret cell death results illuminated by PI in the next section. However, characteristics that impeded resolution of anatomic detail by DIC or prevented broad cellular penetration and nuclear staining of BB presented notable limitations of this approach in the context of *H. contortus* larvae, compared with previous work with *A. suum* [4]. Although DIC coupled with fluorescence microscopy resolved useful anatomic details, this method is cumbersome and unreliable for efficient quantitative assessment of larvae affected in replicates of experimental treatments. In contrast, the inverted microscope supports quantitative assessments of larvae in wells of culture plates, but provides less anatomic detail. Consequently, the two approaches are complementary for extracting information on the larval stages investigated.

### 2.4. Propidium Iodide (PI) Detection of Cell Death in H. contortus xL3 and L4

**Figure 5:**
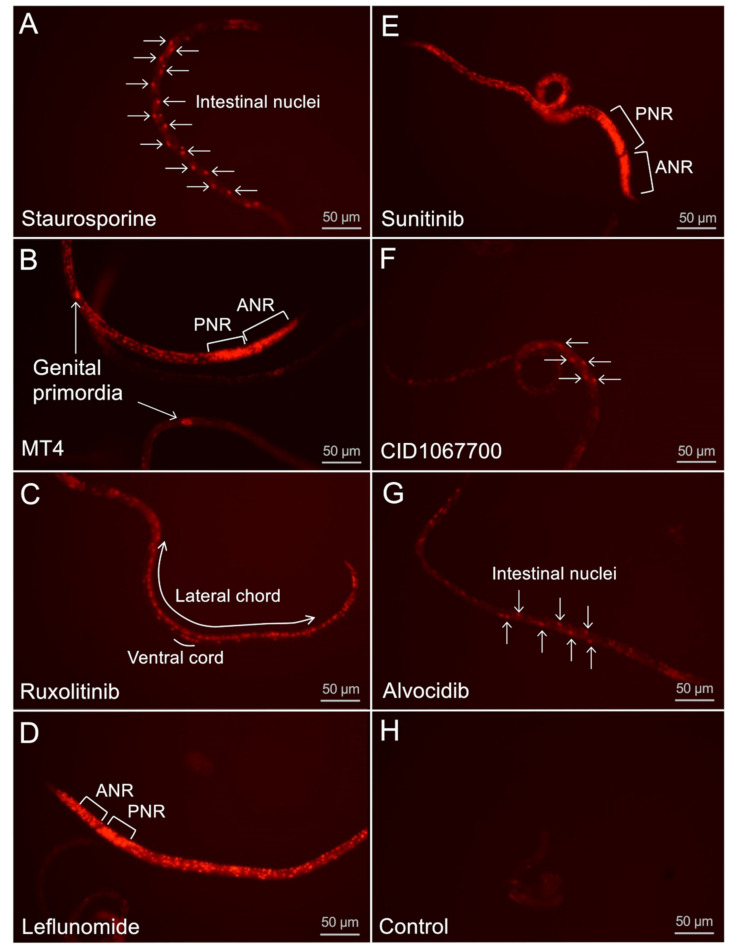
L4-stage *H. contortus* propidium iodide (PI) cell death staining (20× inverted scope) following 2 days of treatment with (**A**) staurosporine (25 µM), demonstrating an intestinal-focused cell death pattern; (**B**) MT4 (500 µM) demonstrating death of genital primordia cells, not visible in BB staining; (**C**) ruxolitinib (500 µM), demonstrating cell death along the lateral line as well as cells in the ventral cord; (**D**) leflunomide (500 µM), demonstrating widespread cell death including staining of both the ANR and the PNR (PNR is not visible in BB live-cell staining); (**E**) sunitinib (500 µM), demonstrating widespread cell death; (**F**) CID1067700 (500 µM), demonstrating an intestinal-focused cell death pattern; (**G**) alvocidib (500 µM), demonstrating staggered intestinal nuclei patterning, and (**H**) controls, demonstrating a lack of cell death in untreated L4 larvae.

As in a previous study in *A. suum* [4], PI staining assays were performed to determine if NIT treatments can induce cell death, to identify the range of cells that can be induced to undergo cell death in larvae (L4), and to compare different NIT characteristics that are identifiable by PI staining. The highest concentration of each NIT used in motility assays (500 μM for all except staurosporine at 25 μM) was used to ensure positive results. Images of PI-stained larvae were recorded after 2 days of treatment for each NIT (Figure 5). In many, but not all cases, the small size of *H. contortus* larvae often compromised resolution and assignment of staining to specific cells and organ systems. Therefore, images presented are an attempt at representing the most common patterns for each NIT. For example, leflunomide induced general PI labeling in most worms (Figure 5D). Although it is difficult to identify many of the cells affected, it seems safe to conclude that leflunomide causes cell death in many, if not all, cells and organs of these larvae, inclusive of neurons posterior to the nerve ring that were refractory to BB labeling in live larvae. Hence, susceptibility to pharmacologic induction of cell death extends to many cells and organs of *H. contortus* L4s. Most other NITs caused more restricted PI staining patterns, several of which identified the staggered appearance of intestinal cell nuclei (Figure 5A,F,G), others included apparent seam cell nuclei, while others produced a PI staining pattern consistent with ventral chord cells (hypodermal, neuronal, or nuclei of both; Figure 5C). Although PI staining of genital primordia was observed in some treatments, this effect was most obvious for MT4-treated larvae (Figure 5B), whereas the broad PI staining in leflunomide treatments often obscured genital primordia for assessment. In contrast with NIT treatments, control larvae generally showed very little background PI staining (Figure 5H).

**Figure 6:**
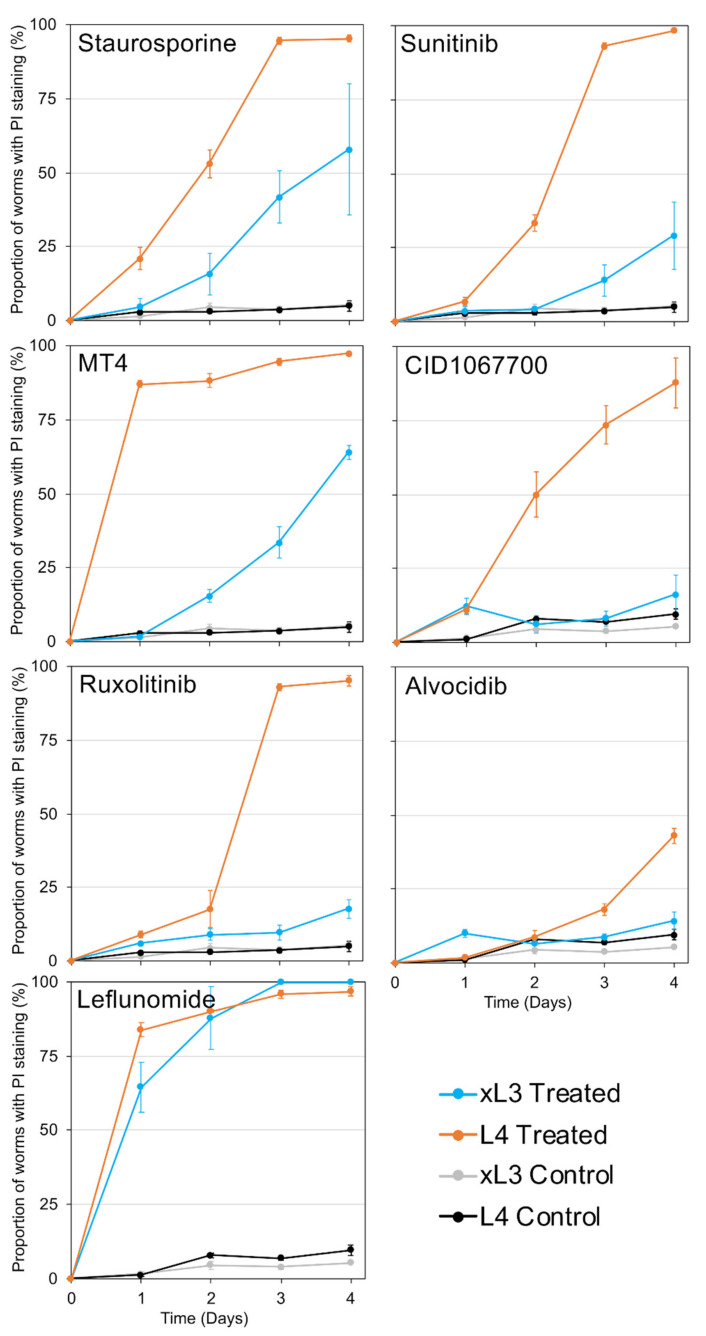
Time-course PI cell death assays for NITs of interest and untreated controls, for xL3 and L4 *H. contortus* larvae. All NITs were applied with a concentration of 500 µM except Staurosporine at 25 µM.

Time course experiments evaluated PI staining in xL3 and L4 treated with NITs (Figure 6), requiring at least five nuclei to be labeled for a positive score regardless of the location of nuclei in the larvae. Use of the inverted microscope to assess labeling of larvae in individual culture wells at 200× provided an efficient means to collect these data, whereas transference to slides and viewing with DIC/fluorescent microscopy was neither reliable nor efficient for this purpose. For xL3, PI labeled larvae generally accumulated to lower levels than L4s, with the possible exception of leflunomide, and only low accumulations were observed with alvocidib, CID1067700, sunitinib, and ruxolitinib, all with means less than 35% by day 4. In contrast, PI-labeled L4 accumulated to high levels with all NIT treatments, except alvocidib, and leflunomide caused the most rapid response with a mean <80% by day 1.

Next, we evaluated the relationship between movement and PI staining in these assays across NITs (Figure S2) and larval stages. While recognizing the lower sensitivity of xL3s indicated in both assays, a general, but not exclusive, observation is that NITs induced immotility that tended to precede detectable PI labeling (points below the diagonal). A delay the in detection of cell death was most extreme for MT4 in xL3. Exceptions to this association included leflunomide (xL3, L4) and MT4 (L4, but not xL3) treatments, both of which caused high inhibition of motility and PI labeling within 24 h. CID1067700 was the only NIT that regularly produced PI labeling at higher levels than immotility for L4, but not xL3. Consequently, the relationship between motility and detection of cell death is complex, and the combination of responses related to immotility, PI detected cell death and cells affected, and larval stage investigated produced unique profiles for each given NIT. The data extends findings on correlation between PI labeling and motility for *H. contortus* larvae [11], and indicate that information gained from the PI cell death assay adds substantial information to that obtainable from motility assays alone. 

### 2.5. Transcriptional Response of H. contortus Larvae to Selected NITs, Differentially Expressed Genes and Enriched Pathways

**Figure 7:**
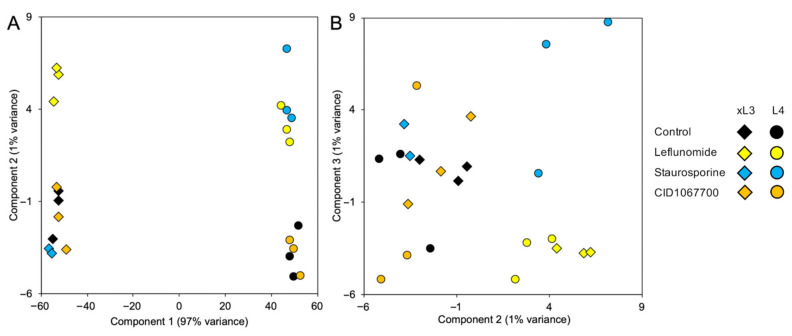
Principal components analysis (PCA) of RNA-seq datasets based on transcriptional profiles for xL3 and L4 *H. contortus* samples. Plots are shown for (**A**) principal components 1 and 2, and (**B**) principal components 2 and 3.

In order to profile the transcriptional responses of xL3 and L4 larval stages of *H. contortus* to NIT exposure, RNA-seq datasets were produced following 2 h of treatment with leflunomide (500 µM), staurosporine (25 µM), CID106770 (500 µM), and DMSO control treatments, in triplicate for both stages. The choice of exposure time and NIT selection was based on maintaining consistency with our similar previous NIT RNA-seq experiments in *A. suum* [4], with the goal of identifying immediate NIT-responsive gene sets before downstream stress-response pathways were activated. Sequencing produced an average of 40.9 million reads, with an average of 73.7% mapping to the genome, following cleaning and processing (Supplementary Table S1A). Principal components analysis (PCA) of RNA-seq datasets indicated substantial transcriptional differences between xL3 and L4 *H. contortus*, regardless of NIT treatment (Figure 7A). Looking at the second and third PCA components, leflunomide treatment produced the most consistent and substantial transcriptional responses across both xL3 and L4, while CID1067700 treatment induced the smallest overall transcriptional changes, consistent with the delayed motility phenotype observed (Figure 3). DEseq2 differential expression analysis also supported these findings, identifying the most genes significantly differentially expressed for leflunomide (380 genes in xL3, 199 genes in L4) and staurosporine (1669 genes in xL3, 129 in L4), while very few genes were differentially expressed for CID106770 (18 genes in xL3, no genes in L4). All gene annotations, read counts, normalized expression values, and differential expression statistics are available in Supplementary Table S1B.

Next, we identified the most significantly enriched KEGG pathways among the differentially expressed genes in both xL3 and L4 stages, using gene set enrichment analysis (GSEA) [18]. Due to the lack of differential expression observed for CID1067700, no pathway enrichment for both larval stages was observed for this NIT. The most significantly enriched pathway among genes upregulated by leflunomide treatment in both stages (Table 1) was “citrate cycle (TCA cycle)”, and the top pathways among the genes downregulated by leflunomide (Table 2) included “Protein processing in endoplasmic reticulum” and “MAPK” signaling. Following staurosporine treatment, the most significantly enriched pathways among the upregulated genes in both stages (Table 3) included “signaling pathways regulating pluripotency of stem cells”, “Phosphatidylinositol signaling system”, and “Inositol phosphate metabolism”, while the most significantly enriched pathways among the downregulated genes (Table 4) included “Fatty acid elongation” and “Ether lipid metabolism”. 

## 3. Discussion

We explored the application of methods established with *A. suum* to rapidly determine cell death in otherwise intact *H. contortus* larvae in culture following treatments with seven different NITs. To the extent accomplished, the application has now been established for representatives of two phylogenetic lineages (*H. contortus*, clade V; *A. suum*, Clade III) of the Nematoda, while also revealing multiple differences in the performance of the assay between the two different parasites, as will be discussed. The term nematode intestinal toxins/toxicants (NITs) reflects the original focus of research to establish practical approaches that kill intestinal cells of parasitic nematodes [3]. However, and not surprisingly, results obtained with *A. suum* and now *H. contortus* indicated that multiple NITs individually cause cell death in many tissues and cells beyond the intestinal tract, which demonstrates broader application of the original research focus. To investigate potential cellular target proteins and early pathways that may converge on cell death pathways, GRPs of *H. contortus* larvae to three selected NITs were evaluated. Some *H. contortus* gene responses resembled those found in *A. suum* and suggested potential cell targets, particularly for leflunomide.

### 3.1. Rapid Determination of Cell Death in H. contortus 

The methods used to detect cell death and assign dead cells to specific cell types or organ systems has clear application to *H. contortus* in otherwise intact L4 and xL3 maintained in culture. The collection of cells documented as undergoing cell death varied among NITs, indicating specificity of outcome attributable to each NIT. For instance, treatments with some NITs led to PI nuclear staining primarily in intestinal cells [4], while for others (for example, leflunomide, sunitinib, and MT4) cell death was detected in many tissues and cells. As with *A. suum*, PI nuclear staining in whole *H. contortus* xL3 and L4 indicates: (1) the ability of a given treatment to induce cell death, for which rapid methods to resolve individual cells in whole worms were only recently described for parasitic nematodes [4]; and (2) the capacity of individual cells to undergo cell death, which, once achieved, is a terminal status and one of interest for anthelmintic research. The very broad extent of PI labeling induced by treatments with leflunomide, sunitinib, and MT4 establishes that many if not all cells of *H. contortus* L4, in particular, are susceptible to pharmacologic inducement of cell death pathways, making knowledge of mechanisms involved relevant to anthelmintic research. Treatment with other NITs cause more restricted PI staining, thus potentially providing experimental tools selective for more restricted cell and organ populations. The intestine, the target tissue for the initial prioritization of the NITs, appears to be more specifically targeted by the most effective NIT, staurosporine, as well as by CID1067700 and alvocidib. That individual cells or organs warrant attention for anthelmintic strategies is supported by evidence that excretory/secretory cells in microfilaria of *Brugia pahangi* are an apparent individual cell target for ivermectin [19], and intestinal cells of multiple parasitic nematodes are crucial anthelmintic targets for benzimidazole anthelmintics [2]. Another notable finding is that some treatments caused apparent neuronal degeneration, which is a more extreme pathologic response than interference with neurotransmission, an endpoint achieved by numerous extant anthelmintics. A similar end point for neurons was noted for *A. suum* [4]. Thus, with specific neurons identified coupled with treatments that cause their degeneration and a means to detect that endpoint, valuable tools are now available to investigate mechanisms involved. Similar logic is applicable to cells identified as undergoing cell death in other organ systems.

However, an important caveat to use of PI on whole nematodes rather than cells in culture relates to potential false negative PI staining. In this case, cells may die but go undetected by PI staining due to the lack of access of PI to the damaged cell in whole worms, as discussed previously [4]. PI must gain access across the cuticle, gut, or other access points in order to stain nuclei of dead cells within the nematode. With this caveat in mind, care is needed relative to questions investigated, experimental designs, and interpretations of results with PI staining in whole nematodes. Equally important, though, is that the caveat does not apply to positive results, which confirms access of PI to the affected cell by virtue of nuclear labeling. With an assay that detects cell death, cellular mechanisms (targets and pathways) responsible for this outcome might be investigated with other treatments (inhibitors) designed to prevent cell death based on targets annotated for NITs, e.g., cell pathways impacted by NITs (RNAseq) and mediators of cell death known in other organisms, including nematodes [20]. A reason for interest in these mechanisms stems from the fact that cell death is pharmacologically targeted in treatments of other diseases [21,22] and might provide useful targets in anthelmintic strategies. 

### 3.2. Comparisons of Microscopy and NIT Responses between H. contortus and A. suum

#### 3.2.1. Cell and Organ Assessments 

Despite this successful application of new methods to *H. contortus*, interspecies comparisons indicated that characteristics of *A. suum* larvae supported better resolution of details on pathologic effects. As one example, better performance was evident in the far more comprehensive live BB staining and DIC resolution of organs and cells in *A. suum* L3 and L4. The number and range of nuclei labeled by BB in live *H. contortus* was more restricted and similar in pattern for both xL3 and L4. Notable absences of, or restrictions on, staining in *H. contortus* larvae included nuclei of cells posterior to the nerve ring, intestinal cells, and many ventral chord nuclei of prospective hypodermal cells and neurons. *A. suum* L3 and L4 both feed, and while only *H. contortus* L4 feed (xL3 do not), restrictions on staining were similar for both *H. contortus* stages. Thus, differences in feeding competence, and internal delivery of BB by ingestion, does not alone explain the differences in BB staining between the two species. The results suggested that unknown physical differences possibly related to cuticular and/or cell membrane constituents differentially impede BB access to specific sites in *H. contortus* compared with *A. suum* larvae. 

Resolution of cell and organ anatomy by DIC was also somewhat compromised in *H. contortus* larvae. BB was used in conjunction with DIC to resolve anatomy with substantial clarity in *A. suum* larvae. Thus, the limited scope of BB staining, light refraction by intestinal inclusions that obscured detail for DIC microscopy, and a perceived less-distinct differentiation of cells by DIC with *H. contortus* larvae together translated into comparatively lower resolution of anatomical structures. Coupled with these challenges, the smaller larvae of *H. contortus* also lessened confidence in assigning PI labeled nuclei to specific cells and organs in many cases (although not all, see Figure 5) when viewed at a magnification conducive for efficient counting of tens of larvae in replicate wells (20× objective, inverted microscope). In sum, while the success in application of the methods to *H. contortus* offers optimism for utility with other parasitic nematodes, the interspecies differences also indicate that specifics of the application could vary considerably among species.

#### 3.2.2. NITs 

We extended the list of NITs to include an inhibitor of p38 MAP kinases (MT4), which inhibited larval movement in *A. suum* and caused demonstrable intestinal cell damage, thus meeting the definition of a NIT established in this species. MT4 also was effective against *H. contortus* in motility and PI nuclear staining assays. That p38 MAP kinases are candidate targets for anthelmintics was also established in *Brugia malayi* [23], but not in relation to specific nematode cells or organs.

The target proteins ascribed for each NIT in mammalian cells are largely distinct among the NITs (summarized in [3]). Detrimental effects resulted from treatment of *H. contortus* larvae by way of immotility and cell death for each NIT, thus suggesting that these outcomes can be initiated through disruption of multiple distinct targets of NITs in nematode cells. A balancing view is that the actual targets of NITs in *A. suum* and *H. contortus* remain unknown and NITs have regularly been used in our experiments at relatively high concentrations to obtain positive results from which to work toward mechanisms of anthelmintic effects. Target proteins of several of the NITs include kinases and elements of signal transduction pathways, and categorically similar inhibitors have shown efficacy against *H. contortus* larvae [24], inclusive of demonstrable phenotypes beyond motility [25]. It is also unclear if the target for an individual NIT is the same among all affected cells, such as those identified by PI staining. Still, differences in PI staining patterns observed among different NIT treatments support that perturbation of multiple different targets can lead to the outcome of cell death in both *H. contortus* and *A. suum*. In this regard, while NITs may or may not represent prospective anthelmintics, it seems clear that they do represent experimental tools to investigate nematode cellular targets and pathways that lead to the anthelmintic outcome of cell death in two phylogenetically distant species, *H. contortus* and *A. suum*. 

#### 3.2.3. Gene Response Patterns

Gene response patterns (GRPs) can provide information relevant to target proteins and pathways influenced by toxic treatments of cells [4]. GRPs to NIT treatments also support that different NITs initiate effects via different targets and cell pathways. For instance, GRPs to selected NITs (leflunomide, staurosporine, and CID1067700) showed marked differences in *A. suum* L4 [4]. 

In humans, leflunomide targets only dihydroorotate dehydrogenase (DHODH) at lower doses, with some inhibition of tyrosine kinases at higher doses [26]. Among the pathways upregulated by leflunomide in both xL3 and L4 of *H. contortus*, the most significant enriched pathway was “citrate cycle (TCA cycle)”, which was also identified as the second-most enriched pathway after 2 h of leflunomide exposure in L3 *A. suum* [4]. DHODH links cellular respiration (including the TCA cycle) with pyrimidine synthesis [27], so this pathway may indicate compensation for leflunomide’s indirect downstream effects. Several pathways were enriched both among the genes most significantly upregulated and among the genes most significantly downregulated with leflunomide, including “Protein processing in endoplasmic reticulum”, a pathway previously shown to be disrupted and differentially expressed in response to leflunomide treatment in cancer cells [28], along with the activation of “MAPK signaling”, which we identified here as being upregulated by both leflunomide and staurosporine, indicating more general NIT responsiveness. The same study [28] also identified enrichment of the Jak-STAT signaling pathway following leflunomide treatment in cancer cells, which was the second-most upregulated pathway in this study. In addition, the pathways “thermogenesis” and “valine, leucine and isoleucine degradation” identified as being upregulated in response to leflunomide were also upregulated in our previous study of L3 *A. suum* [4]. Although no direct link is obvious between these pathways and leflunomide activity, “valine, leucine and isoleucine degradation” may be linked to the described alterations in the TCA cycle, due to its production of propionyl CoA which enters the TCA cycle at the succinyl CoA step [29]. Overall, the pathways identified here indicated that leflunomide has similar transcriptional-level effects on *H. contortus* as for *A. suum*, and as previously described in mammalian cell models. These results, again, are consistent with DHODH as a candidate target whose perturbation by leflunomide might initiate cellular pathways leading to cell death. The ability to induce RNAi with dsRNA treatments (reviewed in [30]) could facilitate investigations on this hypothesis. 

The most significantly enriched KEGG pathway among the genes upregulated by staurosporine treatment (Table 3) was “signaling pathways regulating pluripotency of stem cells”, a mechanism directly supported by previous research showing that staurosporine regulates lineage choice in pluripotent cells by acting as a broad-spectrum inducer of molecular gastrulation [31]. The other pathways only enriched among genes upregulated by staurosporine treatment include “Phosphatidylinositol signaling system” and “Inositol phosphate metabolism”, pathways which were shown to be affected by staurosporine in human platelets through the hydrolysis of phosphatidyl inositol 4,5-bisphosphate [32], as well as “mTOR signaling pathway”, which was previously shown to be affected by staurosporine through the inhibition of phosphorylation of mTOR transcriptional regulators [33]. Interestingly, “synaptic vesicle cycle” was upregulated by staurosporine, which contains the “exocytosis” target pathway for which staurosporine was originally prioritized as a NIT [3]. The pathways enriched only among the genes downregulated by staurosporine (Table 4) include “Fatty acid elongation” and “Ether lipid metabolism”, which may be related to the previously-described differential regulation of sphingolipids in response to staurosporine treatment of cancer cells [34]. Here, we described several staurosporine-regulated pathways in *H. contortus* that are consistent with results from other studies. Nevertheless, the wide range of cellular effects known to be induced by staurosporine complicates the identification of a specific molecular target.

Overall, several NIT-specific and shared GRPs were identified among NITs, which were conserved across both xL3 and L4 stages. These shared pathway responses may reflect more general stress responses, even though the early treatment timepoint (2 h) aimed to minimize these general stress responses and cell death responses, in order to better elucidate the NIT-specific responses. However, since CID1067700 was relatively slow-acting in terms of motility inhibition and cell death (Figure 3 and Figure 5), this 2-h timepoint seemed insufficient to observe a consistent cellular GRP response. The identification of multiple unique cell targets and affected pathways reflects the differences in NIT motility responses, as well as the cell death assays showing unique NIT profiles. It should be noted that the *H. contortus* xL3 larvae for the GRP analysis were obtained from a different laboratory-passaged strain than the rest of the experimentation, but the results obtained here were consistent with our previous *A. suum* study with a similar design, as well as with existing literature describing cellular responses to these NITs, indicating broad phylogenetically consistency in application of methods and responses to NITs. However, the phylogenetic levels at which differences emerge is not known and could include interstrain variability. Overall, our results highlighted that the NITs have unique and complex mechanisms of action that do not simply immediately result in cellular death. 

## 4. Materials and Methods

### 4.1. Ethics Statement 

All animal experiments were carried out under protocols approved by Washington State University Institutional Animal Care and Use Committee, protocol 4097. The protocols meet requirements of AVMA Guidelines for the Euthanasia of Animals: 2013 Edition; Guide for the Care and Use of Laboratory Animals: 2011 Edition, National Research Council, and USA Animal Welfare Act and Animal Welfare Regulations: 2017 Edition (AWA), US Department of Agriculture.

### 4.2. Haemonchus contortus L3, L4 and Adults

*Haemonchus contortus* L3 larvae were obtained from two sources; small batches of laboratory-passaged L3 were purchased from Dr. Ray Kaplan (University of Georgia, Department of infectious diseases, Athens, GA, USA) and used in most experiments presented here, and large batches (1 to 2 million) of laboratory-passaged L3 provided by Zoetis (Dr. Debra Woods, Global Parasitology Research at Zoetis, Kalamazoo, MI, USA) were used to determine gene transcript responses to experimental treatments. Isolates from both sources were designated as susceptible to contemporary anthelmintics. Exsheathed L3 (xL3) were produced by incubation of L3 in 0.125% sodium hypochlorite at 37 °C for twenty minutes. xL3 were washed 5 times in sterile phosphate buffered saline (pH 7.4) by pelleting larvae at 600× *g* for five minutes, discarding the supernatant, and then adding PBS for the next rinse. After the final rinse, pelleted xL3 were resuspended in RPMI medium (R8758, Sigma-Aldrich, St. Louis MO) containing 20% fetal bovine serum, 100 units penicillin, and 100 μg Streptomycin per mL (P0781, Sigma-Aldrich, St. Louis, MO, USA) and incubated at 38 °C with 20% CO_2_. L4 were generated by incubation of xL3 in this conditioned media for 7 days. Acquisition of L4 characteristics (well-defined mouth) was observed by this time in a mean 95.3% (±1.3%) of larvae. Culture volumes were 100 μL in 96 well plates (3595, Costar, Corning Inc., Corning, NY, USA) and 1 mL in 24 well plates for applications in this research. 

### 4.3. Ascaris suum L3, L4

*A. suum* lung-stage L3 were obtained as described before [3]. Briefly, infective larvated eggs were used from batches generated from previous studies [3,4]. Then, 4000 eggs were used to orally infect New Zealand white rabbits (5.5 to 6.5 weeks old, Western Oregon Rabbit Company, Philomath, OR, USA) and L3 were collected from the lungs by lavage. Larvae were settled by gravity and then washed in 3 sequential 50 mL volumes of warm PBS followed by 3 sequential 15 mL volumes, with intervening gravity sedimentation and discard of supernatant PBS. Extracted and cleaned larvae were then suspended in RPMI medium (R8758, Sigma-Aldrich, St. Louis, MO, USA, containing 10% swine serum, 100 units penicillin and 100 μg Streptomycin/mL; P0781, Sigma-Aldrich, St. Louis, MO, USA) and then, if used for testing as L3, dispensed into wells of 96-well plates (3595, Costar, Corning Inc., Corning, NY, USA, triplicate wells for each treatment), with a total volume of 100 μL culture medium containing treatments with diluent (DMSO, 922401 JT Baker, Center Valley, PA, USA) at 1%. L3 were then incubated at 37 °C in 5% CO_2_ for times prescribed for each experimental design. Alternatively, to obtain L4, L3 were incubated in 1 mL culture medium contained in a 15 mL polypropylene tube (62.554.100, Sarstedt, Newton, NC, USA) with a loosened screw cap for 3 days, and medium was replaced daily with fresh medium. L3 molted to L4 by day 3. L4 generated in this way were dispensed into wells of 96 well plates used for experiments described here and incubated as for L3. 

### 4.4. Fluorescent Marker Labeling

To determine the ability of xL3 and L4 *H. contortus* to ingest fluorescent probes, two function-based fluorescent probes were used in feeding assays: (1) beta-ala,lys-AMCA (BAL-AMCA), 200 μM (BP0352, BioTrend, Zurich, Switzerland) [12]; and (2) DQ Green-BSA (D-12050, Molecular Probes, Eugene, OR, USA), 100 μg/mL [13]. L3 and L4 were cultured in the presence of these probes for 4 h prior to assessment of ingestion. To assess staining of nuclei by bisbenzimide (BB, 10 µg/mL, H 33258, Sigma-Aldrich, St. Louis, MO, USA) live xL3 and L4 were incubated with BB in standard culture conditions for 16 h prior to assessment by fluorescent microscopy. To assess cell death in whole xL3 and L4, propidium iodide (PI, P4170, Sigma-Aldrich, St. Louis, MO, USA) was used at 100 μM, and added to larval cultures 4 h prior to the addition of experimental treatments. Preincubation leading to ingestion of PI was intended to maximize access of tissues to the fluorescent probes in presence of various treatments. 

### 4.5. Microscopy and Imaging

Fluorescent labeling of larvae in wells of 96 well plates was monitored using a Nikon Diaphot 300 inverted microscope equipped with epifluorescence capabilities: UV-2A filter (blue), BB, BAL-AMCA; G1A (red filter), PI; B2A (green filter), DQ-BSA) and recorded with a Nikon D5100 digital camera. Most observations were made using the 20× microscope objective (200× magnification). To better resolve cells and organs in which BB-stained nuclei reside, larvae were transferred from wells and placed onto agar pads on glass microscope slides and viewed using the 60× microscope objective (600× magnification) on a Nikon Optiphot compound microscope equipped with differential interference contrast (DIC) filters, epifluorescence capabilities, and a Nikon D5100 digital camera. To optimize resolution of DIC/fluorescence details, images were captured in movie mode, and then selected screen shots were copied and used to produce final digital images. 

### 4.6. NIT Treatments

A set of chemicals identified as nematode intestinal toxins/toxicants (NITs) [3] were used in experiments here and included: alvocidib (S1230), sunitinib (S7781), Selleckchem Houston, TX, USA; CID1067700 (SML054), leflunomide (L5025), p38 MAP kinase inhibitor IV (a.k.a. MT4, which was used in this paper, SML0543) Sigma-Aldrich, St. Louis, MO, USA; ruxolitinib (tlrl-rux), InvivoGen, San Diego, CA, USA; staurosporine (S-9300), LC Laboratories, Woburn, MA, USA. DMSO was used as diluent for chemical treatments, except propidium iodide was solubilized in PBS, and concentrations were adjusted to achieve a maximum 1% DMSO after all components were included for each experiment. Control wells were adjusted according to diluent used for treatment wells. With the exception of staurosporine, treatments were initiated at 500 μM (staurosporine, 25 μM) to better ensure the possibility of achieving a positive result with PI labeling based on previous experience [3]. Dose response experiments to determine effective dose 50 (IC_50_) were conducted using two-fold serial dilutions over a range of 5 concentrations. IC_50_ values were calculated using AAT Bioquest (Sunnyvale, CA, USA) IC_50_ calculator (https://www.aatbio.com/tools/IC50-calculator/ (accessed on June 17 2021)).

### 4.7. Motility PI labeling Assays

xL3 and L4 were dispensed into triplicate wells of 96 well plates containing more than twenty larvae each, in 100 μL of medium containing treatments. Movement was scored using the inverted microscope, and 200× magnification. Plates were agitated manually to encourage movement of larvae and larval movement was scored if observed during an approximate 45 sec observation period. Mean motility for each treatment was determined for each day over the course of 4 days. The setup for PI labeling assays was identical, except that wells also contained PI and BB, and PI labeling was scored with the inverted fluorescent microscope using 200× magnification. A conservative PI scoring system was used in that more than 5 nuclei labeled with PI in a given larva were required to be scored positive. The mean number of PI positive larvae was determined for each treatment, each day, over a course of days.

### 4.8. RNA Extraction, RNA-seq and Differential Expression Analysis

For RNAseq analysis (see below), approximately 80,000 *H. contortus* xL3 or L4 were aliquoted into each of triplicate wells in a 48 well plate and 1 mL of media for each treatment. The treatments included leflunomide (500 μM), staurosporine (25 μM), CID1067700 (500 μM), or diluent (1% DMSO) followed by incubation for 2 h, then rinsing with three 1 mL volumes of PBS (7.4), withdrawing excess PBS and mixing larval pellets with 50 μL TRIzol (Invitrogen/Life Technologies, Carlsbad, CA, USA), and then freezing samples at −80 °C until processed for RNA. RNA was extracted from pellets of untreated and treated larval *H. contortus* frozen in Trizol (described under Section 2.5) for RNAseq analysis by homogenizing larval pellets as they thawed using a microfuge pestle, and then processing TRIzol extracts according to the manufacturer’s instructions. Ethanol pellets of isolated RNA were shipped to Washington University for further processing and sequencing. RNA-seq analysis was performed as previously described for *Ascaris suum* [4]. Briefly, cDNA libraries were prepared from RNA samples using the Clontech SMARTer universal low-input RNA kit to maximize yield and processed cDNA was sequenced on the Illumina NovaSeq S4 platform (paired-end 150bp reads). Trimmomatic v0.36 [35] was used to trim adapters, and the STAR aligner [36] (v2.7.3a; 2-pass mode, basic) was used to map RNA-seq reads to the *H. contortus* genome assembly (PRJEB506.WBPS14 [37]), which were then counted for every annotated protein-coding gene in the assembly using featureCounts [38]. DESeq2 [39] was used to perform differential expression comparisons, comparing each NIT the control samples collected for both xL3 and L4-stage larvae. Principal components analysis (PCA) was carried out using DESeq2 output (default settings). The raw RNA-seq read files (fastq) are accessible on the NCBI Sequence Read Archive (SRA [40], BioProject PRJNA264197), and complete sample metadata, read counts, normalized expression values, and accession information are provided in Supplementary Table S1. 

*H. contortus* genes based on the PRJEB506.WBPS14 annotation and assembly [37] were assigned functional annotations using both InterProScan v5.42 [41] to identify gene ontology assignments as well as InterPro domains, and GhostKOALA v2.2 [42] to assign KEGG annotations. Potentially secreted proteins were identified using both SignalP v5.0 [43] for signal peptides and transmembrane domains, and SecretomeP v2.0 [44] to identify proteins with non-classical secretion sequences. Gene set enrichment analysis (GSEA [18]) was performed, based on DESeq2 differential expression results, to test for enrichment among non-human KEGG pathway annotations using WebGestalt v2019 [45] (FDR-adjusted *p* ≤ 0.05, minimum 5 genes differentially expressed). The input values for GSEA analysis were the ranks (largest value to smallest) of the genes when ranked based on (a) differential expression *p* value from DESeq2 analysis, following log transformation, where upregulated genes were calculated using negative log, and downregulated genes were calculated using positive log, and (b) Log_2_ fold change value when *p* values were tied. The top 15 most significantly enriched KEGG pathways shared between both xL3 and L4 for each NIT were identified (according to FDR-adjusted *p* values). “Normalized enrichment scores” shown in Table 1, Table 2, Table 3 and Table 4 were the primary statistic calculated by Gene Set Enrichment Analysis to evaluate results, and represent the degree to which a gene set is overrepresented at the top or bottom of a ranked list of genes, normalized for differences in gene set sizes (since smaller gene sets are more likely to all be close to the top of a list by random chance).

## 5. Conclusions

This study presented an interdisciplinary approach designed to intersect (i) methodology that comprehensively resolved, in whole unfixed parasites, NIT-induced pathology among most cells and organs in *H. contortus*, and (ii) interrogation of transcriptional responses to NIT treatments, resulting in the identification of their putative molecular targets and pathways. The assays directly identified the constellation of cells and organ systems that undergo cell death with respect to each of multiple chemical toxins/toxicants. Susceptibility to inducible cell death adds rationale to investigate the cells and organs identified in anthelmintic research, and advances established here provided new experimental methods to do so. We also identified, for some NITs, cellular pathways and molecular targets that are potential antecedents to irreparable pathologic outcomes. Our original derivation of NITs involved a *de novo* approach [3] which, when building on methodology we have developed [4] and integrated with results described here, establish a unique and useful experimental system for the purposes of discovery and detailed analysis of anthelmintic compounds and investigation of mechanisms of their toxicity. Ultimately, knowledge of mechanisms that actually mediate cell death coupled with pathways whose disruption initiate death processes hold much potential for application in anthelmintic research. The results shown here confirmed cross-species efficacy of the NITs, which were computationally predicted to have broad efficacy across nematodes. In addition to their previously demonstrated efficacy against *A. suum*, an important parasite of humans, our results demonstrated efficacy against species of a great veterinary importance, the blood feeding parasitic nematode of small ruminants *H. contortus*. In the future, the efficacy of these NITs may also be tested to other parasitic nematodes of economic and clinical importance, including as plant or aquatic parasitic nematodes, given that here, we demonstrated consistent efficacy spanning multiple nematode clades.

## Figures and Tables

**Table 1:** Top 15 KEGG pathways most significantly upregulated in both xL3 and L4 *H. contortus* in response to leflunomide treatment.

KEGG Pathway		xL3		L4		Also Lower with LEF	Also Higher with STU
		Normalized Enrichment Score	FDR-Adjusted *p* Value	Normalized Enrichment Score	FDR-Adjusted *p* Value		
20	Citrate cycle (TCA cycle)	2.01	5.3 × 10^−5^	1.93	5.5 × 10^−4^	-	-
4630	Jak-STAT signaling pathway	1.92	3.0 × 10^−4^	1.69	6.0 × 10^−3^	-	-
4714	Thermogenesis	2.25	<1 × 10^−5^	1.57	1.6 × 10^−2^	-	-
3050	Proteasome	1.77	1.5 × 10^−3^	1.80	4.1 × 10^−3^	-	-
280	Valine, leucine and isoleucine degradation	1.76	1.7 × 10^−3^	1.75	4.0 × 10^−3^	-	-
4141	Protein processing in endoplasmic reticulum	2.06	<1 × 10^−5^	1.89	1.4 × 10^−3^	Y	-
4137	Mitophagy-animal	1.92	3.2 × 10^−4^	2.07	<1 × 10^−5^	Y	-
3040	Spliceosome	1.92	3.4 × 10^−4^	2.37	<1 × 10^−5^	Y	-
4530	Tight junction	2.14	<1 × 10^−5^	1.88	1.7 × 10^−3^	-	Y
4068	FoxO signaling pathway	1.94	2.4 × 10^−4^	1.77	4.2 × 10^−3^	-	Y
4371	Apelin signaling pathway	1.90	3.2 × 10^−4^	1.68	6.4 × 10^−3^	-	Y
4010	MAPK signaling pathway	1.81	1.0 × 10^−3^	1.78	4.0 × 10^−3^	-	Y
4723	Retrograde endocannabinoid signaling	1.96	2.8 × 10^−4^	1.96	2.1 × 10^−2^	-	Y
4152	AMPK signaling pathway	1.80	1.1 × 10^−3^	1.63	1.0 × 10^−2^	Y	Y
4212	Longevity regulating pathway-worm	1.83	7.7 × 10^−4^	1.60	1.3 × 10^−2^	Y	Y

**Table 2:** Top 15 KEGG pathways most significantly downregulated in both xL3 and L4 *H. contortus* in response to Leflunomide treatment.

KEGG Pathway		xL3		L4		Also Higher with LEF	Also Lower with STU
		Normalized Enrichment Score	FDR-Adjusted *p* Value	Normalized Enrichment Score	FDR-Adjusted *p* Value		
4961	Endocrine and other factor-regulated calcium reabsorption	1.77	3.7 × 10^−3^	1.65	1.0 × 10^−2^	-	-
360	Phenylalanine metabolism	1.67	8.9 × 10^−3^	1.76	3.5 × 10^−3^	-	-
3018	RNA degradation	1.86	1.7 × 10^−3^	1.55	2.3 × 10^−2^	-	-
4136	Autophagy-other	1.66	9.6 × 10^−3^	1.83	1.7 × 10^−3^	-	-
4150	mTOR signaling pathway	1.66	9.5 × 10^−3^	1.71	5.7 × 10^−3^	-	-
561	Glycerolipid metabolism	1.62	1.3 × 10^−2^	1.69	6.7 × 10^−3^	-	-
4721	Synaptic vesicle cycle	1.53	2.3 × 10^−2^	1.65	9.6 × 10^−3^	-	-
4137	Mitophagy-animal	1.74	5.1 × 10^−3^	1.80	2.3 × 10^−3^	Y	-
4212	Longevity regulating pathway-worm	1.61	1.3 × 10^−2^	1.51	3.2 × 10^−2^	Y	-
4152	AMPK signaling pathway	1.59	1.4 × 10^−2^	1.54	2.5 × 10^−2^	Y	-
3022	Basal transcription factors	2.22	<1 × 10^−5^	1.50	3.5 × 10^−2^	-	Y
250	Alanine, aspartate and glutamate metabolism	1.88	1.5 × 10^−3^	1.46	4.6 × 10^−2^	-	Y
3010	Ribosome	1.54	2.1 × 10^−2^	2.77	<1 × 10^−5^	-	Y
3040	Spliceosome	1.95	5.3 × 10^−4^	2.46	<1 × 10^−5^	Y	Y
4141	Protein processing in endoplasmic reticulum	1.57	1.7 × 10^−2^	1.98	2.1 × 10^−4^	Y	Y

**Table 3:** Top 15 KEGG pathways most significantly upregulated in both xL3 and L4 *H. contortus* in response to Staurosporine treatment.

KEGG Pathway		xL3		L4		Also Higher with LEF
		Normalized Enrichment Score	FDR-Adjusted *p* Value	Normalized Enrichment Score	FDR-Adjusted *p* Value	
4550	Signaling pathways regulating pluripotency of stem cells	1.82	1.1 × 10^−3^	1.60	1.7 × 10^−2^	-
4926	Relaxin signaling pathway	1.78	1.5 × 10^−3^	1.60	1.7 × 10^−2^	-
4070	Phosphatidylinositol signaling system	1.83	9.4 × 10^−4^	1.55	2.0 × 10^−2^	-
4012	ErbB signaling pathway	1.73	2.3 × 10^−3^	1.61	1.7 × 10^−2^	-
4150	mTOR signaling pathway	1.89	5.1 × 10^−4^	1.53	2.3 × 10^−2^	-
562	Inositol phosphate metabolism	1.64	5.2 × 10^−3^	1.70	1.6 × 10^−2^	-
4721	Synaptic vesicle cycle	1.95	2.7 × 10^−4^	1.47	3.2 × 10^−2^	-
4310	Wnt signaling pathway	1.78	1.5 × 10^−3^	1.54	2.1 × 10^−2^	-
4212	Longevity regulating pathway-worm	2.01	2.1 × 10^−4^	1.67	1.5 × 10^−2^	Y
4010	MAPK signaling pathway	1.93	2.8 × 10^−4^	1.66	1.5 × 10^−2^	Y
4068	FoxO signaling pathway	2.00	1.8 × 10^−4^	1.61	1.7 × 10^−2^	Y
4723	Retrograde endocannabinoid signaling	2.00	1.9 × 10^−4^	1.56	2.0 × 10^−2^	Y
4371	Apelin signaling pathway	2.12	<1 × 10^−5^	1.53	2.3 × 10^−2^	Y
4152	AMPK signaling pathway	1.91	4.0 × 10^−4^	1.56	2.0 × 10^−2^	Y
4530	Tight junction	2.08	<1 × 10^−5^	1.44	3.8 × 10^−2^	Y

**Table 4:** Top 15 KEGG pathways most significantly downregulated in both xL3 and L4 *H. contortus* in response to Staurosporine treatment.

KEGG Pathway		xL3		L4		Also Lower with LEF
		Normalized Enrichment Score	FDR-Adjusted *p* Value	Normalized Enrichment Score	FDR-Adjusted *p* Value	
650	Butanoate metabolism	2.00	1.8 × 10^−4^	1.61	1.7 × 10^−2^	**-**
3060	Protein export	2.12	<1 × 10^−5^	1.53	2.3 × 10^−2^	**-**
3008	Ribosome biogenesis in eukaryotes	1.78	1.5 × 10^−3^	1.60	1.7 × 10^−2^	-
4744	Phototransduction	1.91	4.0 × 10^−4^	1.56	2.0 × 10^−2^	-
62	Fatty acid elongation	1.83	9.4 × 10^−4^	1.55	2.0 × 10^−2^	-
270	Cysteine and methionine metabolism	1.73	2.3 × 10^−3^	1.61	1.7 × 10^−2^	-
3420	Nucleotide excision repair	1.76	1.8 × 10^−3^	1.58	1.9 × 10^−2^	-
565	Ether lipid metabolism	1.89	5.1 × 10^−4^	1.53	2.3 × 10^−2^	-
30	Pentose phosphate pathway	2.08	<1 × 10^−5^	1.44	3.8 × 10^−2^	-
4146	Peroxisome	1.64	5.2 × 10^−3^	1.70	1.6 × 10^−2^	-
4141	Protein processing in endoplasmic reticulum	2.01	2.1 × 10^−4^	1.67	1.5 × 10^−2^	Y
3040	Spliceosome	1.93	2.8 × 10^−4^	1.66	1.5 × 10^−2^	Y
250	Alanine, aspartate and glutamate metabolism	2.00	1.9 × 10^−4^	1.56	2.0 × 10^−2^	Y
3022	Basal transcription factors	1.78	1.5 × 10^−3^	1.63	1.6 × 10^−2^	Y
3010	Ribosome	1.82	1.1 × 10^−3^	1.60	1.7 × 10^−2^	Y

## Data Availability

The RNA-seq data presented in this study are openly available in NCBI Sequence Read Archive (SRA), BioProject PRJNA264197. Complete sample metadata, mapped read counts, normalized expression values, and accession information are provided in Supplementary Table S1.

## Supplementary Materials

The following are available online at https://www.mdpi.com/article/10.3390/ph14070598/s1, Figure S1: IC50 values for all NITs in L4-stage H. contortus, at days 1 to 4; Figure S2: Association between motility inhibition and PI staining for each of the seven NITs. All NITs were applied with a concentration of 500 µM except Staurosporine at 25 µM. PI was applied 4 h pre-treatment and included throughout the treatment period; Supplementary Table S1: RNA-seq data, including (A) Metadata and read count statistics for all RNA-seq samples and (B) Functional annotations, mapped RNA-seq read counts, FPKM relative expression values, and DESeq2 differential expression results for all genes and all RNA-seq samples.
